# Osteotomies and Total Knee Arthroplasty: Systematic Review and Meta-Analysis

**DOI:** 10.3390/life12081120

**Published:** 2022-07-26

**Authors:** Kulinski Krzysztof, Ewa Trams, Stanislaw Pomianowski, Rafal Kaminski

**Affiliations:** Department of Orthopaedics and Trauma Surgery, Centre of Postgraduate Medical Education, Professor A. Gruca Teaching Hospital, Konarskiego 13, 05-400 Otwock, Poland; k.kulinski@o2.pl (K.K.); ewa.trams@gmail.com (E.T.); spom@spskgruca.pl (S.P.)

**Keywords:** osteotomy, total knee arthroplasty, TKA, TTO, epicondylar osteotomy, reduction osteotomy

## Abstract

Total knee replacement (TKA) is a frequent modality performed in patients with osteoarthritis. Specific circumstances can make it much more difficult to execute successfully, and additional procedures such as osteotomy may be required. The aim of this study was to perform a meta-analysis and systematic review of osteotomies combined with TKA. Methods: In June 2022, a search PubMed, Embase, Cochrane, and Clinicaltrials was undertaken, adhering to PRISMA guidelines. The search included the terms “osteotomy” and “total knee arthroplasty”. Results: Two subgroups (tibial tubercle osteotomy and medial femoral condyle osteotomy) were included in the meta-analysis. Further subgroups were described as a narrative review. The primary outcome showed no significant difference in favor to TTO. Secondary outcomes showed improved results in all presented subgroups compared to preoperative status. Conclusion: This study showed a significant deficit of randomized control trials treated with osteotomies, in addition to TKA, and a lack of evidence-based surgical guidelines for the treatment of patients with OA in special conditions: posttraumatic deformities, stiff knee, severe varus, and valgus axis or patella disorders.

## 1. Introduction

The concept of deformity correction has been known since the time of Hippocrates and has evolved through the centuries from osteoclasia—the breaking of a deformed bone—to the first surgical osteotomy performed by John Rhea Barton in the USA in 1835 [1,2,3]. Extra-articular deformity is defined by mechanical axis deviation (MAD), joint orientation angles (MPTA—medial proximal tibial angle, LDFA—lateral distal femoral angle, PPTA—posterior proximal tibial angle, and PDFA—posterior distal femoral angle), and joint line convergence angle (JLCA). The center of rotation of angulation (CORA) indicates the point of angular deformity. Importantly, intra-articular deformities are often presented by joint incongruency [4,5]. The CORA point should be determined before any osteotomy for proper surgery planning, because it determines the distance of the deformity from the joint [6]. Nowadays, osteotomies around the knee are widely used for the treatment of knee osteoarthritis, posttraumatic malalignment, or congenital deformities. A surgeon can perform an osteotomy as a single procedure, as well as with concomitant soft tissue surgery, articular cartilage regenerative procedure, or ligament reconstruction [7,8]. The principles of osteotomies were described by Paley, who defined standard lower limb alignment and joint orientation, as well as malalignment and malorientation in different planes. He described radiographic assessment of deformities and referred to each joint, as well as total knee arthroplasty (TKA), associated with malalignment [5]. The mechanical and anatomical principles of osteotomy are of great concern in order to achieve appropriate alignment and joint congruency after surgery [8,9]. The most frequently performed osteotomy is the medial open-wedge high tibial osteotomy (HTO), which is for the treatment of early stage osteoarthritis [10], and the tibial tubercle osteotomy (TTO), which is for recurrent patella dislocation or patellofemoral arthritis [11]. Additionally, osteotomies are of common use during total knee arthroplasty (TKA): TTO as an approach for revision surgery or epicondylar osteotomy for TKA balancing [12].

To our knowledge, this is the first systematic review and meta-analysis that considers all osteotomies, in addition to TKA. Every study performed prior was concerned with a specific type of osteotomy (tibial tubercle and medial/lateral sliding osteotomy) or clinical problem (varus/valgus malalignment, patellofemoral disorders, and tibial malunion). In our study, we would like to summarize the outcomes and indications for all osteotomies concomitant with TKA. This review could lead to future prospective studies and expand our knowledge concerning those types of surgeries.

## 2. Materials and Methods

### 2.1. Search Strategy

This systematic review and meta-analysis was constructed according to the Preferred Reporting Items for Systematic Reviews and Meta-analyses—the 2020 PRISMA statement [13]—and was registered in the PROSPERO International prospective register of systematic reviews (ID 310946). In June 2022, a comprehensive published literature search through PubMed, Embase, Cochrane Database of Systematic Reviews, and Clinicatrials.gov was performed. The references to the investigations found in this search were cross-referenced to identify additional pertinent studies not identified in the original searches. All searches were performed for studies concerning osteotomy performed around the knee joint. The searches were performed by combining the following keywords: “osteotomy” and “total knee arthroplasty”. The search was restricted to the English language.

### 2.2. Inclusion and Exclusion Criteria

This review included all clinical trials concerning osteotomies on the distal femur or proximal tibia. All types of clinical trials meeting the following criteria were included in the analysis: English language, human subjects, paper published in a peer-reviewed journal, and full text available. Due to the small number of randomized clinical trials identified, we included in the review a comparative retrospective or non-randomized studies. The exclusion criteria included all animal studies, basic scientific investigations, case reports, review articles, expert opinions, letters to editor, studies without control groups, technical notes, papers not peer-reviewed, papers not in English, and trials evaluating osteotomy unrelated to TKA. We excluded 12 articles in French, German, and Chinese due to the possibility of “Tower of Babel bias”. The investigations included in this study were independently reviewed by two orthopedic surgeon authors for inclusion and exclusion criteria. Studies included in the analysis were divided into the following subgroups: tibial tubercle osteotomy and medial femoral condyle osteotomy. Studies which did not meet those criteria were included in our study as a narrative review in the following subgroups: tibial tubercle osteotomy, medial femoral condyle osteotomy, lateral femoral condyle osteotomy, femoral osteotomy, and tibia osteotomy.

### 2.3. Selection and Data Collection

**Figure 1:**
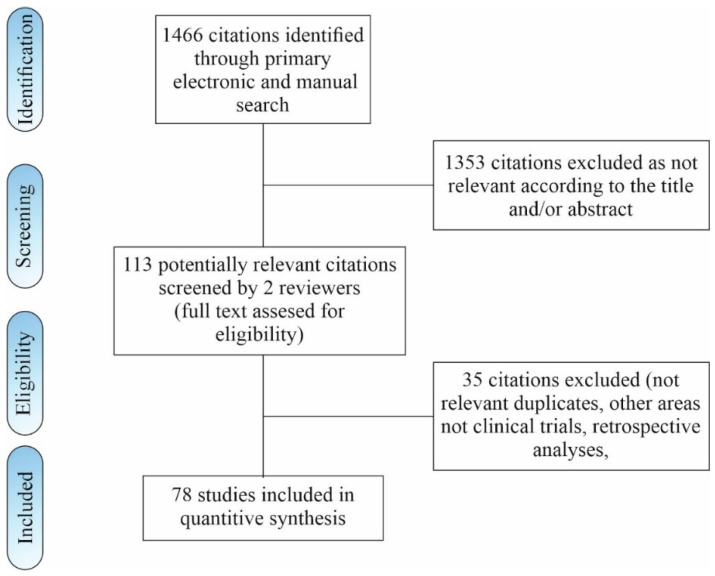
Flowchart of study inclusion.

Two authors (K.K. and E.T.) independently searched databases, screened the resulting titles, and extracted the abstracts. The full-text articles were accessed for the articles, which were adjustable by topic for the senior author (R.K.). Two reviewers (R.K. and E.T.) extracted the data from the articles. At every search stage, the extracted data were cross-checked, and any disagreements were discussed and settled by a third author. A literature search through an electronic database identified a total of 1466 records according to the search algorithm. One hundred and thirteen abstracts of the remaining articles were assessed for the eligibility criteria. Thirty-five of them were excluded. Fifteen clinical trials were included in this review and meta-analysis, and sixty-three were included as a narrative review due to the important overall results of the main topic. The literature search flowchart is shown in Figure 1.

### 2.4. Data Items

For each study included in the analysis, the following data were extracted by two independent reviewers: authors, year of publication, type of knee lesions, details of interventions in the study, sample size (randomized and analyzed), outcome measurements, follow-up period, main results, and type of adverse events included in the publication. Each study’s level of evidence was examined and evaluated based on criteria established by OCEBM Levels of the evidence working group [14]. The measure of final stage treatment effect was the mean together with the standard deviation for continuous outcome measures. When studies reported other measures (e.g., median) and other dispersion measures, such as the standard error (SE) of the mean or 95% confidence interval (CI) of the mean, range, or interquartile range (IQR), we calculated the SD in order to perform the relevant meta-analytical pooling according to References [15,16].

### 2.5. Types of Interventions

We compared TTO or epicondylar osteotomy with the following:

- Quadriceps snip approach;
- Anterior medial knee approach;
- Soft tissue release balancing.

### 2.6. Outcomes

The primary outcomes included the following:

- Pain—measured by a standard validated pain scale, e.g., Visual Analogue Score (VAS), EQ-VAS score, or Numeric Rating Scale (NRS);
- Functional measurements by any standard validated scale, such as The Western Ontario and McMaster Universities Osteoarthritis Index (WOMAC), Knee Society Score (KSS), Hospital for Special Surgery knee rating scale (HSS), and Oxford Knee Score (OKS);
- Range of motion (ROM);
- Limb alignment assessed as femorotibial angle;
- Adverse events—if multiple time points were reported within our timeframes, we extracted the last time point (e.g., if data were reported at six weeks, three months, six months, and one year, we extracted outcomes at one year).

### 2.7. Study Risk of Bias Assessment

Revised Cochrane risk-of-bias tool was used to evaluate risk. Disagreement in the risk of bias assessment was resolved by consensus and, if necessary, by the opinion of a third reviewer. A study was deemed to be one of the following:

- “Low risk”—all items were scored as “low risk”;
- “Moderate risk”—up to two items were classified as “high risk” or “unclear risk”;
- “High risk”—if more than two items were scored as “high risk”.

We have presented our assessment of risk of bias by using two “Risk of Bias” summary figures for every subsection of the manuscript.

### 2.8. Effect Measures and Synthesis Methods

The study weight was calculated by using the Mantel–Haenszel method. We assessed statistical heterogeneity by using Tau^2^ or Chi^2^, df, and I^2^ statistics. The I^2^ statistic describes the percentage of total variation across trials that is due to heterogeneity. In the case of low heterogeneity (I^2^ < 40%), studies were pooled by using a fixed-effects model; otherwise, a random-effects analysis was made.

### 2.9. Statistical Analysis

Qualitative statistical analysis and meta-analysis were performed by using R™ software 4.2.0 and REVMAN 5.4.1 [17,18]. The *p*-values <0.05 were considered to be significant.

## 3. Results

### 3.1. Femoral Shaft Osteotomy

Eight case series included in our study concerned distal femoral osteotomy as an additional procedure to TKA [19,20,21,22,23,24,25,26] (Table 1). Five of them presented one-stage total knee arthroplasty with distal femoral osteotomy for the correction of extra-articular deformity with good results in the last follow-up (4 to 10 years of follow-up) [19,20,21,22,25]. Five of them included post-traumatic patients with femoral fractures malunion (in total, 19 patients) [19,20,21,22,25]: constitutional in three patients, secondary after femoral osteotomy with overcorrection in three patients [22], and one with hypophosphatemic rickets [25]. Clinical and functional outcomes were shown in KSS [19,21,25,26], HSS [22], Kujala, and Oxford Knee Score [23], with significant increases at the last follow-up. Six studies also showed improvement in ROM post-surgery (range 85–107) [20,22,23,26,27]. The surgical technique was different in every study. One study presented the case of a patient with habitual patellar dislocation after TKA treated with biplanar closed wedge distal femoral osteotomy with concomitant medial patellofemoral ligament reconstruction. The two-year follow-up reported good outcomes [23]. The case presentation of a 19-year-old patient with juvenile rheumatoid arthritis showed bilateral osteotomy with TKA for treatment of flexion contractures. The first knee was treated with distal femoral resection, with conversion to hinged arthroplasty, and the second by femoral shortening osteotomy, with resurfacing TKA. Function outcomes as well as ROM showed notable improvements. Better results were observed after a shortening femoral osteotomy than for distal femoral resection [27]. Walter et al. described the case of a patient with achondroplasia treated with a bilateral rotating hinged implant with closed-wedge femoral osteotomy with good results—pain relief and greater range of motion [26].

**Table 1:** Distal femoral osteotomy around TKA.

Author	Year	Study Design	No. of Patients	Follow-Up	Clinical Outcomes	Radiological Outcomes	Complications	Fixation	LOE
Catonne [19]	2019	prospective	6	10 years	KSS (21 to 77.5), KSF (25 to 83); ROM (95 to 107)	HKA (178–182), FMA (89–93), TMA (89–91)	1 deep vein thrombosis; 1 stiffness—flexion 70 (110 finally)	Long uncemented extension stem + posterior-stabilized implant	IV
Fan [21]	2014	case	1	2 years	Rom 0–110, KSF 80	X-ray	none	TKA + long Gamma 3 nail	IV
Kitchen [27]	2015	case	1 patient with juvenile rheumatoid arthritis	2 years	ROM improved to 20–75 18–85	X-ray	none	Cemented stem	IV
Lonner [25]	2000	retrospective	11 patients	46 months	KSS (10 to 87), KSF (22 to 81), ROM (56 to 89), flexion contracture (19 to 2)	Mechanical axis (2); union	1 nonfatal postoperative pulmonary embolism; 1 osteotomy did not heal; 2 required improve ROM; 1 required removal of TT screws	Press-fit stem/blade-plate/retrograde nail + press-fit condylar/Legacy/Osteonics	IV
Rueda [20]	2016	case	1 hemophilic type A	8 years	Rom (0–70 to 0–90)	X-ray	X-ray—loosening in the tibial component	Revision stem NexGen (Zimmer Biomet) + 4.5 mm locking-compression-plate-dynamic-compression-plate	IV
Saito [23]	2020	case	1 with patellar dislocation	24 months	ROM 0–130, Kujala functional score (24 to 58), OKS (28 to 40)	Patella tilt angle (40 to 20); bisect offset (121 to 60); FTA (172); HKA (2)	none	Mpfl reconstruction + biplanar closed wedge distal femoral osteotomy	IV
Sun [22]	2021	retrospective	7	91 months (38–104 months)	HSS (45 to 90), collateral ligament laxity, ROM (70 to 105)	Mechanical axis deviation (MAD), mLDFA, mMPTA, JLCA	1 nonunion; 1 intraoperative split fracture of distal femur; 1 wound exudation	Long cemented stem	IV
Walter [26]	2017	case	1 with achondroplasia	12 months	KSS (4–12 to 78–79), ROM (0–60/75 to 0–75/85)	X-ray EOS	none	NexGen (Zimmer) + LC-plate with lag screws	IV

LOE—level of evidence; ROM—range of movement, KSS—Knee Society Score, KSF—Knee Society Score functional score, OKS—Oxford knee scale, HSS—Hospital for Special Surgery knee rating scale, FTA—femorotibial angle, HKA—hip–knee–ankle angle, Kujala—patellofemoral score, MAD—mechanical axis deviation, mLDFA—mechanical lateral distal femoral angle, mMPTA—mechanical proximal tibial angle, JLCA—joint line convergence angle, TT—tibial tubercle.

### 3.2. Lateral Aspect of a Knee

#### Lateral Femoral Condyle Osteotomy

Six studies presented outcomes after lateral femoral condyle osteotomy for the correction of severe valgus deformity in TKA (Table 2). All patients require specific ligament balancing surgery to achieve proper correction and knee stability [28,29,30,31,32]. Preoperative valgus deformity ranged from 10 to >20 degrees, and it was reported in three articles [28,31,32,33] and described as a fixed valgus deformity (the valgus deformity was not passively correctable) in two articles [29,30]. Four of the studies were prospective [28,29,30,32], and two of them were retrospective [31,33], but none of them had a control group. All authors decided to perform lateral femoral sliding osteotomy in one-stage surgery with TKA. Only one accomplished the surgery without screw fixation—a lateral collateral ligament complex was attached to a sliver of bone [33]. Other authors, after performing osteotomy, used two or three screw (cannulated or cancellous) fixations. A total of 203 patients included in all trials presented significantly improved outcomes in the last follow-up (median 1 to 5 years): OKS [32,33], KSS [28,29,30,31,32], KSF [31,32], WOMAC [29], HSS [29], and Stability Score [28]. All studies after surgery had an X-ray for radiological measurements of correction. One case report also showed good results for a patient with progressive varus deformity after lateral epicondylar osteotomy in the TKA procedure [34].

### 3.3. Medial Aspect of a Knee

#### 3.3.1. Medial Femoral Condyle Osteotomy

Six studies described medial epicondyle osteotomy in TKA (Table 3) [35,36,37,38,39,40]. Three randomized control trials (RCTs) were included in the meta-analysis [36,37,38]. Mirzatolooei et al. evaluated outcomes in 14 patients with bilateral varus deformity in OA. They compared osteotomy technique vs. medial collateral ligament (MCL) release by pie-crusting or subperiosteally release in another leg. After the 12-month follow-up, they found non-significant differences in ROM, VAS, WOMAC, Oxford scores, and flexion contracture when comparing these two techniques [37]. Twenty patients included in another retrospective study compared distal release of MCL and medial epicondylar osteotomy for ligament balancing in TKA for medial contracture in varus knee. They found non-significant differences in functional and clinical scores, as well as in flexion contracture. The only significant difference was shown in valgus stress radiograms [38]. Mou et al. compared constrained arthroplasty to posterior stabilized arthroplasty and medial femoral epicondyle up-sliding osteotomy in patients with severe (>30 degree) valgus deformity. Fifty-three patients were enrolled in this study, and they were monitored for more than 50 months. The KSS Function Score and ROM significantly increased in both groups post-operatively but were non-significant in the post-operative group compared to patients preoperatively. Only the Hospital for Special Surgery knee rating scale showed significant difference in favor of the osteotomy group (*p* < 0.05) [36].

**Figure 2:**
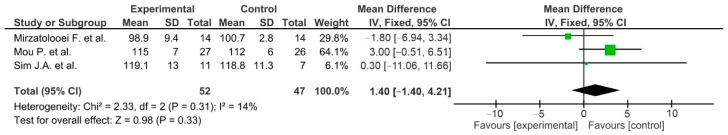
Forest plot for range of movement (ROM) (CI, confidence interval; IV, inverse variance; and SD, standard deviation).

**Figure 3:**
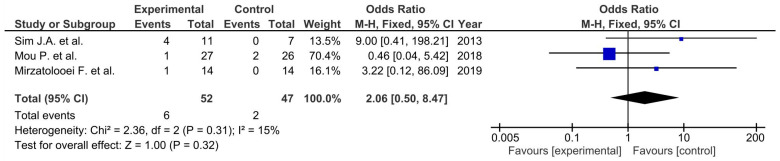
Forest plot for adverse events (CI, confidence interval).

**Figure 4:**
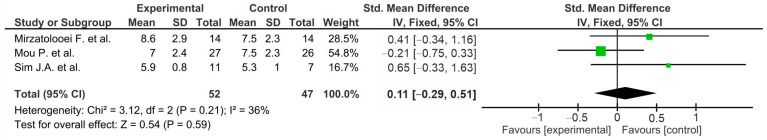
Forest plot for femorotibial angle (CI, confidence interval; IV, inverse variance; and SD, standard deviation).

**Figure 5:**
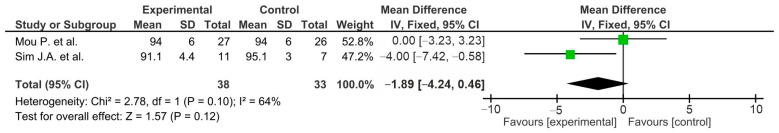
Forest plot for KSS function (CI, confidence interval; IV, inverse variance; and SD, standard deviation).

**Figure 6:**
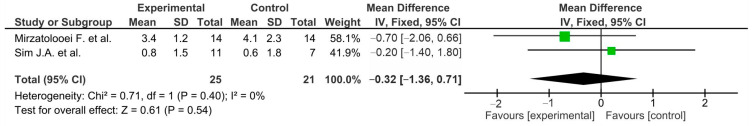
Forest plot for flexion contracture (CI, confidence interval; IV, inverse variance; and SD, standard deviation).

**Figure 7:**
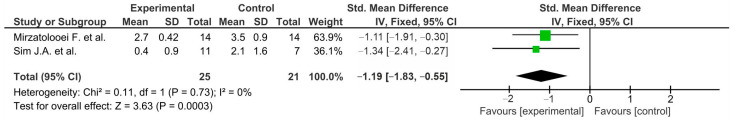
Forest plot for medial joint opening (CI, confidence interval; IV, inverse variance; and SD, standard deviation).

Pooled estimated for these studies showed non-significant differences in favor of the control group (*p* > 0.05) in ROM (Figure 2), unwanted events (Figure 3), and the femorotibial angle (Figure 4) [36,37,38]. Two of them showed non-significant differences in favor of the osteotomy group in regard to the KSS function score (Figure 5) (*p* = 0.12) [36,38]. Moreover, similar outcomes were found in flexion contractures (*p* = 0.54) in favor of osteotomy (Figure 6). Only medial joint opening showed significant differences for pooled estimated studies in favor of the osteotomy group (*p* = 0.0003) [37,38] (Figure 7). 

**Table 2:** Lateral condyle femoral osteotomy around TKA.

Author	Year	Study Design	No. of Patients	Follow-Up	Clinical Outcomes	Radiological Outcomes	Complications	Fixation	LOE
Brilhault [30]	2002	prospective	13	56 months	KSS (32 to 88); KSF (45 to 73)	FTA (191 to 180); patellar tilt, lateral shift, Insall–Salvati; Caton–Dechamps	4 deep-vein thrombosis, 1 superficial wound problem, 1 pseudoarthrosis of sliding osteotomy	3.5 mm screw	IV
Chen [34]	2015	case	1	1 year	ROM 0–105	FTA 145 to 176	none	3 screws	IV
Hadjicostas [28]	2008	prospective	15	28 months	KSS (37 to 90); KSF (40 to 90); instability score (12 to 21.3); flexion (85 to 105)	Valgus (21 to 0.5); internal rotation (9.5 to 0.5)	2 lateral releases; 1 early superficial hematoma	bicortical screw	IV
Li [31]	2018	retrospective	25	3.3 years	KSS (36.5 to 89.1)	Valgus (21 to 0.5); HKA (202.7 to 180.4), aLDFA (74.6 to 82.4), aLPTA (82.7 to 89.6), FTEA (84.2 to 89.6), IS (0.95 to 0.9),	1 transient numbness in the peroneal nerve area, 1 wound exudation	3 cancellous screws	IV
Raut [33]	2018	retrospective	23	5 years	OKS 43; Arc of movement (110)	FTA (20 to 4)	none	none	IV
Scior [32]	2018	prospective	98	4.5 years	OKS (41.2 to 21.3); KSS to (35.9 to 84.9); KSF (56 to 83.1)	FTA (14.9 to 6.4); HKA (189.7 to 179.4); ADLF (76.4 to 83.7); MPTA (91.4 to 89.6)	1 displacement; 1 infection, 1 periprosthetic fracture, 1 aseptic loosening of tibial component, 1 instability; 2 capsule rapture	2 cannulated 4.5 mm screws	IV
Strauch [29]	2012	prospective	27	1 year	WOMAC (41.9 to 20); AKSS (87.9 to 157.5); patella score (12.5 to 24.5); ROM (118.8 to 119.4)	FTA (17.7 to 7.2); patellar tilt; patellar shift; ADFL (76.1 to 83.0); MPTA (91 to 89.8); tibial slope (7.9 to 4); HKA (191 to 180.3)	none	2 cannulated 4.5 mm screws	II

LOE—level of evidence; ROM—range of movement, WOMAC—Western Ontario and McMaster Universities Osteoarthritis Index, KSS—Knee Society Score, KSF—Knee Society Score functional score, AKSS—Knee Society Score activity score, OKS—Oxford knee scale, FTA—femorotibial angle, HKA—hip–knee–ankle angle, mLDFA—mechanical lateral distal femoral angle, mMPTA—mechanical proximal tibial angle, TT—tibial tubercle.

**Table 3:** Medial condyle femoral osteotomy around TKA.

Author	Year	Study Design	No. of Patients	Follow-Up	Clinical Outcomes	Radiological Outcomes	Complications	Fixation	LOE
Mihalko [39]	2013	retrospective	102	63.2 months	ROM (80 to 103.6); flexion contracture (21.8 to 2.2); KSS (29.4 to 94.5); KSF (31.4 to 78.3)	Varus (11.8 to 6)	none	-	IV
Mirzatolooei [37]	2019	prospective	14	1 year	ROM (98.9); WOMAC (39.8); OKS (88.3); Flexion contracture (3.4); Vas 39.5	Varus (22.6 to 7.5)	1 nonunion	2 pins	III
Mou [36]	2018	prospective	53	50 months	KSF (33 to 94); HSS (30 to 91); ROM (83 to 115)	VA (31.1 to 7); HKA (179.9); FTA (173)	Wound problem, peroneal nerve injury, patellar tracking dysfunction, infection, pulmonary embolism; knee instability, recurrent valgus deformity, implant loosening, osteolysis, motion deficit	4.5 mm hollow screws	II
Mou [35]	2018	prospective	26	6 years	KSF (33 to 94); HSS (30 to 91); ROM (84 to 116)	HKA (203.5 to 179.9); FTA (147.9 to 172.9); CHA (106.8 to 89.8); PAA (95.4 to 90.2); VA (32.1 to 7.3)	1 periprosthetic femoral fracture;	4.5 mm hollow screws	IV
Sim [38]	2013	retrospective	9	46.5 months	KSS (35.9 to 91.1); KSF (33.6 to 88.2); flexion contracture (11.8 to 0.8); ROM (103 to 119)	FTA (11 to 5.9)	4 fibrous union	Suture Ethibond	III
Sim [40]	2018	retrospective	61	50.6 months	KSS (35.3 to 89.1); KSF (48.7 to 88.6); flexion contracture (8.5 to 1); ROM (112 to 118.9)	FTA (10.4 to 5.5); varus (16.7 to 1)	22 fibrous union	6.5 mm cancellous screw/non absorbable sutures	IV

LOE—level of evidence; ROM—range of movement, WOMAC—Western Ontario and McMaster Universities Osteoarthritis Index, KSS—Knee Society Score, KSF—Knee Society Score functional score, OKS—Oxford knee scale, HSS—Hospital for Special Surgery knee rating scale, FTA—femorotibial angle, HKA—hip–knee–ankle angle, TT—tibial tubercle.

Two further studies included in this review described medial femoral epicondyle up-sliding osteotomy for correcting valgus deformity and to balance soft tissues. Mou et al., in a prospective study, described good outcomes in 26 patients in 54 +/− 18 months follow-up, but with no control group. Valgus angles were measured over 20 degrees, with knees belonging to the Krackow II classification, and outcomes were measured on the Hospital for Special Surgery knee rating scale (HSS), knee society function score, and range of motion [35]. Malhaiko et al. used 10 cadaver knees—5 for standard MCL release group after TKA and 5 for an epicondylar osteotomy group. They observed significantly greater laxity at 60 and 90 degrees of flexion for the osteotomy group and also significantly greater laxity at full extension, and 90 flexion included internal and external rotation. They retrospectively analyzed 102 patients with a minimum 1-year follow-up, using the standard medial parapatellar approach and subperiosteal release of MCL with increased clinical and functional outcomes in all cases. They also stated that there is no need for this type of exposure, and if this procedure is performed, it should be performed with extreme care, PCL preservation and the use of a constrained implant [39]. Sim el al. described medial epicondylar osteotomy for correcting varus TKA in a retrospective study of 54 patients in a 2-year follow-up. KSS, ROM, and radiological outcomes significantly improved compared to preoperative. They also observed 39 osseous union and 22 fibrous union, with no differences between the bone and fibrous groups [40].

#### 3.3.2. Medial Tibial Reduction Osteotomy

Five studies described TKA with reduction osteotomy in medial side of tibia for gap balancing in varus knee (Table 4) [41,42,43,44,45]. Two RTC designed by Ahn et al. showed better results for the osteotomy group compared to the control group. The first trial compared vertical osteotomy (bony resection of proximal medial tibia) to medial soft tissue release. The most important finding showed reduced operation time (mean 96.9 min for osteotomy vs. 116.2 min for control). There were no significant differences in ROM, HSS, and mediolateral gap in 0 and 90 degrees. Only the 130 degree gap ratio was significantly smaller for the osteotomy group (*p* = 0.0001) [42]. The second study compared reduction osteotomy with pie-crusting for gap imbalance over 3 mm. They found better overall success in the osteotomy group (*p* = 0.007)—five failures vs. seventeen failures in the control group, and a significant difference in the change of medial gap in knee extension balancing (*p* < 0.001). However, the change of the medial gap in knee flexion balancing was significantly better for the pie-crusting group (*p* < 0.001) [41].

Three further studies showed a correlation between the mean correction and the size of the bony resection [43,44,45]. Two of them were prospective [44,45], and in two, computer navigation was used [43,44]. Mullaji et al. described a predictable 1 degree of correction for every 2 mm of bone resected [44]; Krackow et al. showed similar results—mean correction of 0.45 degree for every 1 mm. They also found improvement in KSS and KSF, as well as in the lower extremity activity scale [43]. Niki et al. showed a 1.7-degree correction for 4 mm and 2.8-degree correction for 8 mm bony resection. Additionally, KSS and KFS improved in 3.3 months of follow-up [45].

**Table 4:** Medial tibia reduction osteotomy around TKA.

Author	Year	Study Design	No. of Patients	Follow-Up	Clinical Outcomes	Radiological Outcomes	Complications	Fixation	LOE
Ahn [42]	2013	prospective	27	6 months	HSS (63 to 92.9); ROM (123.75 to 134.45)	Tibio-femoral medial–lateral gap ratio	none	-	II
Ahn [41]	2016	prospective	106	1 month	Flexion contracture (5.9); ROM (121.5)	Varus (10.1 to 0.6)	5 Flexion gap imbalance	-	II
Krackow [43]	2014	retrospective	35	32.8 months	KSS (38 to 89.2); KSF (51.45 to 76.55); LEAS (8 to 9.68)	Varus (9.47 to 0.65)	1 infection	-	IV
Mullaji [44]	2013	prospective	71	-	-	Varus (14 to 3.5)	-	-	IV
Niki [45]	2015	prospective	36	3.3 years	KSS (91.8); KSF (78.3)	FTA (5.9)	9 trabecular metal components	-	IV

LOE—level of evidence; ROM—range of movement, KSS—Knee Society Score, KSF—Knee Society Score functional score, HSS—Hospital for Special Surgery knee rating scale, FTA—femorotibial angle, HKA—hip–knee–ankle angle, TT—tibial tubercle.

### 3.4. Tibial Tubercle Osteotomy

Twelve studies were included in the meta-analysis (Table 5) [46,47,48,49,50,51,52,53,54,55,56,57]. Follow-up ranged from 1 to 15 years; all except three studies [54,56,57] were prospective and consider TTO in both revision and primary TKA. Four of them compared quadriceps sniff with the tibial tubercle osteotomy approach for TKA [47,48,55,58], two lateral subvastus approach with TTO vs. medial parapatellar approach [49,51], and another six medial parapatellar vs. lateral parapatellar approach with TTO [50,52,53,54,56,57]. Clinical and functional outcomes improved in all studies when compared to preoperative data.

Barrack et al., in a multicenter study, showed equivalent results between groups in KSS and patellofemoral questionnaires, a significantly greater arc of motion in the quadriceps group and difficulty with kneeling and stooping in the TTO group [46]. Sun et al. showed no significant differences in KSS, HSS, WOMAC, flexion contracture, and maximal flexion in second-stage revision for infected TKA [55]. Di Benedetto also showed no differences in the ROM and KSS score between the groups [47]. On the other hand, Bruni et al., in their randomized prospective study, showed significantly increased mean knee flexion and KSS score and incidence of extension lag significantly lower for the TTO group in infected knees (*p* < 0.05) [48]. TTO compared with the lateral subvastus approach also showed no significant differences in the WOMAC, KSS, ROM [49,51], and VAS scale [49]. Four complications occurred in the TTO group—three tibial plateau fractures and one wound discharge [51]. A significant increase in lateral patellar subluxation in the standard medial parapatellar approach (*p* = 0.034) was observed [49]. Comparing the lateral with the TTO and standard medial parapatellar approach, three studies showed non-significant differences in outcomes: HSS [57], ROM, KSS [52,53,57], and VAS [52,53]. Schiapparelli et al., in computer tomography measurements, showed no significant differences between the groups in terms of the limb axis [54]. However, Hirschmann et al. showed better KSS (*p* = 0.0009), VAS (*p* = 0.0001), and flexion (*p* = 0.027) in the TTO group [50]. Moreover, Vandeputte et al., in a 2-year follow-up in patients after revision TKA with pseudopatella baja, presented significantly increased clinical KSS (*p* = 0.03); however, functional KSS did not increase significantly (*p* = 0.2) [56].

**Figure 8:**
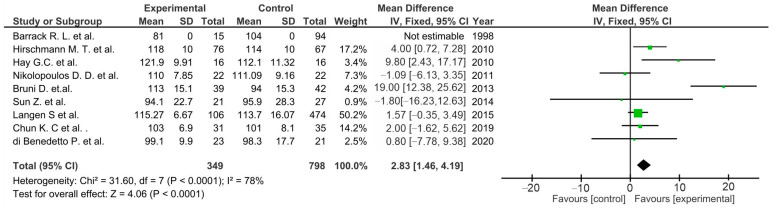
Forest plot for ROM in TTO patients (CI, confidence interval; IV, inverse variance; and SD, standard deviation).

**Figure 9:**
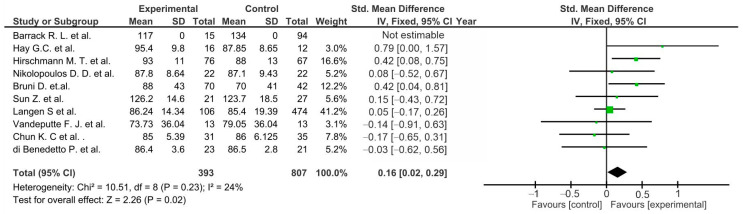
Forest plot for KSS clinical score (CI, confidence interval; IV, inverse variance; and SD, standard deviation).

**Figure 10:**
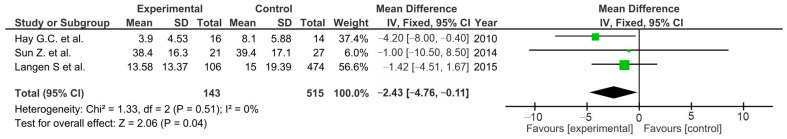
Forest plot for WOMAC (CI, confidence interval; IV, inverse variance; and SD, standard deviation).

Most of the presented studies showed no significant differences between the control and experimental groups. However, pool estimates for studies in the following subgroups showed significantly increased outcomes in favor of the TTO group: ROM (*p* < 0.0001) [46,47,48,49,50,51,52,55,57] (Figure 8), KSS clinical score (*p* = 0.02) [46,47,48,49,50,51,52,55,56,57] (Figure 9), and WOMAC (*p* = 0.04) (Figure 10) [49,51,55].

**Figure 11:**
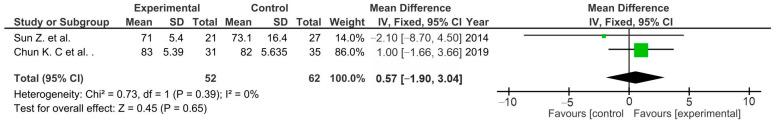
Forest plot for HSS (CI, confidence interval; IV, inverse variance; and SD, standard deviation).

**Figure 12:**
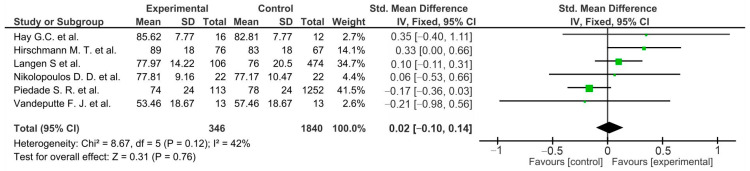
Forest plot for KSS functional score (CI, confidence interval; IV, inverse variance; and SD, standard deviation).

**Figure 13:**
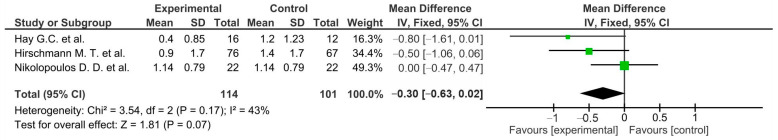
Forest plot for VAS (CI, confidence interval; IV, inverse variance; and SD, standard deviation).

**Figure 14:**
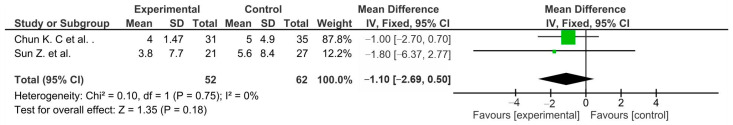
Forest plot for flexion contracture (CI, confidence interval; IV, inverse variance; and SD, standard deviation).

**Figure 15:**
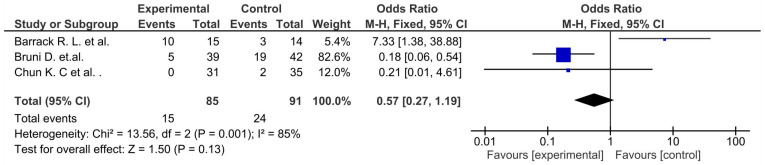
Forest plot for presence of extension lag (CI—confidence interval).

**Figure 16:**
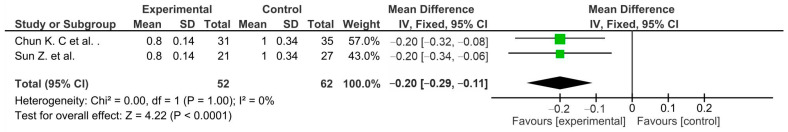
Forest plot for Insall–Salvati ratio (CI, confidence interval; IV, inverse variance; and SD, standard deviation).

**Figure 17:**
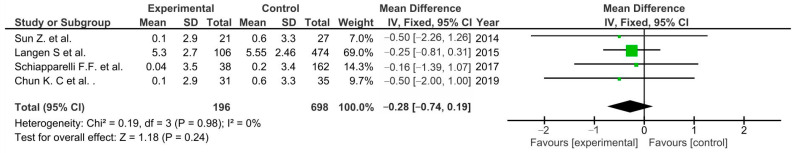
Forest plot for femorotibial angle (CI, confidence interval; IV, inverse variance; and SD, standard deviation).

**Figure 18:**
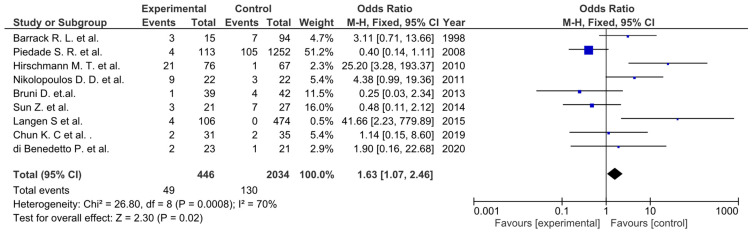
Forest plot for adverse events (CI, confidence interval).

Non-significant differences were observed for the HSS (*p* = 0.65) [55,57] (Figure 11), KSS functional score (*p* = 0.76) (Figure 12) [49,50,51,52,53,56], and VAS score (*p* = 0.07) (Figure 13) [49,50,52], as well as flexion contracture (*p* = 0,18) (Figure 14) [46,48,57] and extension lag (*p* = 0.13) (Figure 15) [55,57]. The Insall–Salvati ratio (*p* < 0.0001) (Figure 16) [55,57] and femorotibial angle (Figure 17) (*p* = 0.24) showed differences in favor of the TTO group. Unwanted events presented a significant difference (*p* = 0.02) in favor of the control group (Figure 18) [46,47,48,50,51,52,53,55,57].

Twenty-one articles were excluded from the meta-analysis of TTO because they did not include a control group (Table 5) [5,58,59,60,61,62,63,64,65,66,67,68,69,70,71,72,73,74,75,76,77,78]. For reattachment osteotomy, most authors used cerclage wires [56,64,67,68,69,70,73,74,75,77], the second most common type of fixation were screws [62,63,65,66,70,71,76] and only two studies reported suture repair [72,78]. TTO approach was applied in both primary TKA, for knees with severe valgus deformity [69,73,76,77], patella disease [62,65,74] or revision surgery (aseptic or septic) [63,64,65,66,67,68,70,71,72,73,75,78]. Eight studies presented 100% bone healing after osteotomy at the last follow-up [62,65,66,70,71,77,78], another nine reported cases of malunion, however single cases were mentioned (1–4 patients) [64,67,68,69,72,73,74,75,76]. Clinical and functional outcomes presented in articles improved significantly from baseline scores.

Abbas et al., in a study that included 159 patients, observed no complication of extensor mechanism after revision TKA [78]. Young et al., in both revision and primary TKA, showed improvement in KSS (*p* < 0.0001) and ROM. However, six patients had persistent extensor lag, and two sustained extensor mechanism disruption [73]. Moreover, Le Moulec showed a significantly increased ROM (*p* < 0.0001) after Chevron osteotomy in revision TKA [75]. Furthermore, Chalidis et al. (*p* < 0.001) [70] and Biggi et al. (*p* < 0.0001) reported increases in KSS and the VAS scale. There was also one extension lag and three cases with flexion lag [71]. Another retrospective study of non-correctable valgus deformity presented significant improvements in knee extension (*p* = 0.002), flexion (*p* = 0.006), KSS (*p* = 0.001), and WOMAC (*p* = 0.001) [70]. In a study with two-stage revision of periprosthetic infection, KSS and ROM significantly increased, but there were 10 reinfections [68]. Punwar et al., after two-stage revision, showed 14 of 16 infections eradicated with no extensor lag and improvement in the ROM and Oxford Knee Score [66]. Apostopoulos et al. showed improvement in the International Knee Society Score (*p* < 0.05) and correction of axis deviation (average 23 degree). The post-operation angle between the mechanical and anatomical axis ranged from 2 to 7 degrees in 22 patients [77]. Eid et al. presented a correction of valgus deformity and knee stiffness in patients with rheumatoid arthritis, with improvement in their HSS score (*p* < 0.0001) [76]. Vives-Barquiel performed TTO for treatment stiffness after TKA with patella baja. A significant improvement in KSS, WOMAC, and VAS was noted [74]. Price et al. performed Fulkerson osteotomy due to chronic patella dislocation after TKA, with an improvement in KSS and ROM in a 2-year follow-up [62]. Moreover, Ries et al. used TTO for extensor mechanism patella realignment for patella instability in TKA, with an increased ROM [65,67].

Another series of four cases analyzed tibial tubercle osteotomy in TKA; three of them, as an approach in revision or primary total knee arthroplasty [58,59], presented with severe intra-articular contracture [60]. Bruce et al. used three cerclage wires for reattachment of osteotomy and observed fixed flexion deformity in three cases. No severe complications were noted; however, there was one instance of radiographic evidence of proximal migration of osteotomy, but with no symptoms [58]. Moreover, Maruyama reported no complications in a 2-year follow-up after surgery. Cortical or cancellous screws were used to reattach the osteotomy [59]. One patient was treated with TTO after multifocal limb reconstruction with 45 degrees of varus deformity. Fibular and closing-wedge osteotomies were also performed [60]. On the other hand, Nakajima et al. described TTO for the treatment of patellar subluxation after TKA. After Elmslie–Trillat procedures and extensive lateral release after a 1-year follow-up, they observed good outcomes (no complaints regarding the knee pain, and a ROM of 0–120), along with no further patellar dislocations (no maltracking or subluxation) [61].

### 3.5. Tibial Shaft Osteotomy

Five case studies, including two prospective [79,80] and two retrospective [81,82] studies, presented TKA with tibia shaft osteotomy (Table 6) [79,80,81,82,83,84,85,86,87]. Three cases presented closing-wedge osteotomy [83,84,86], and two cases presented open-wedge osteotomy [85,87]. Grzelecki et al. described osteotomy in patients with multiaxial deformity in the course of multiple hereditary osteochondromas. They used semi-constrained condylar knee prosthesis with long stems, presenting a significant increase in KSS clinical and functional scores after 1 year of follow-up [83]. Shibano et al. showed significant improvement in KSS clinical and functional scores in a 2-year follow-up after semi-constrained TKA with long stems for treatment of a patient with valgus knee deformity after HTO [86]. Hosokawa et al. had a patient with malunion after tibial plateau fractures. A long-stem tibia TKA implant with a one-third tubular plate was used for the correction. The plate had to be removed at 17 months due to infection, but with no further complications. After 2 years, they observed no pain, full bone union, and a ROM of 0–125° [84]. Ucan et al. presented a case of spontaneous osteonecrosis of the varus knee treated by unicondylar knee replacement with biplanar ascending proximal osteotomy. At the 5-year follow-up, the Oxford scale, as well as the IKDC, increased significantly [87]. The final case presented complications after an input press-fit extension stem in a patient with a varus knee after closed fracture. After 5 months, osteomyelitis occurred, and the patient needed two-stage reimplantation with the Illizarov technique. After 2 years, a callus bridge was observed [85]. Three studies of 48 patients showed good results for treated patients with tibia deformity [79,80,81]. Catonne et al., in a 9-year follow-up, observed a significantly increased IKS score (*p* < 0.0001) and ROM. They used a long-stemmed posterior stabilized tibial implant with a screw, plate, or staple for osteotomy fixation for 13 and 10 cases of open- and closed-wedge osteotomy, respectively [79]. Two studies showed a significant increase in the clinical and functional knee score (*p* < 0.05) after TKA with HTO [80,81]. One study presented seven patients with recurrent patellar dislocation and severe external tibial torsion (>45 degrees). Ramaswamy et al. used a posterior stabilized, stemmed tibial component and two AO cancellous screws and washers. In a 47-month follow-up, they showed significant improvement in the KSS clinical and functional score (*p* = 0.0001), Q-angle (*p* = 0.002), and mean arc of flexion (*p* < 0.0001) [82].

### 3.6. Risk of Bias

**Figure 19:**
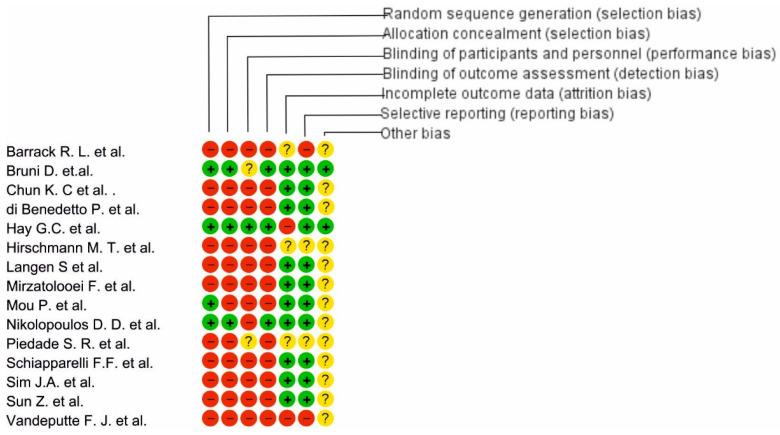
Risk of bias analysis for osteotomy around a knee.

A majority of the studies had a high risk of bias overall. The odds ratio, test for overall effect, and heterogeneity were not estimable for pooled studies. Only one study was in low risk of bias for all applicable domains [48]; two studies had only one high risk of bias [49,52]. In another seven studies, we saw increased risk of bias: they had two in the low-risk domain and four with a high risk of bias [37,38,47,51,54,55,57]. One study had an equal risk of bias, 3 in high and 3 in low risk [36]; and some studies had a high risk of bias [46,50,53,56,88,89,90,91,92,93,94] (Figure 19).

**Table 5:** Tibial tubercle osteotomy around TKA.

Author	Year	Study Design	No. of Patients	Follow-Up	Clinical Outcomes	Radiological Outcomes	Complications	Fixation	LOE
Abbas [78]	2016	retrospective	159	22 months	-	Time to union (11 weeks) 100%	6 proximal migration, 11 fragmentation	Ethibond suture	IV
Apostolopoulos [77]	2010	prospective	24	11.5 years	KSS (44 to 91); ROM (96 to 110);	Valgus (23 to 5.5)	1 proximal migration, 1 deep venous thrombosis, 7 hematoma	3 wire loops (2 patients 2 cortical screw)	IV
Barrack [46]	1998	prospective	15	30 months	KSS (77 to 117); arc of motion (73 to 81)	X-ray	none	Luque wires/screws	III
Di Benedetto [47]	2020	prospective	23	21.5 months	KSS (86.4); ROM (99.1)	Full-length weightbearing radiographs	1 reinfection;	AO laces	III
Biggi [71]	2018	retrospective	79	7.4 years	KSS (40.7 to 75); ROM (78.7 to 95); VAS (7.9 to 3.8)	Bone healing 2.4 months	4 painful hardware, 3 late periprosthetic infection, 1 extension lag, 3 flexion lag	3.5 mm cortical screws	IV
Bruce [58]	2000	prospective	9	3 years	HSS (43.6 to 79.2); ROM (59.5 to 78)	Union (8 weeks proximal; 24 weeks distal)	3 fixed flexion deformity; 2 proximal migration	3 cerclage wires (1 patient 3 screws)	IV
Bruni [48]	2013	prospective	81	12 years	KSS (11 to 88), ROM (113), extension lag (5 to 0)	X-ray	1 Deep venous thrombosis	two stage Revision TKA, fixation with wires	I
Chalidis [70]	2009	retrospective	74	49 months	ROM (80 to 95); arc of motion (60 to 95); extensor lag (10 to 5); flexion contracture (10 to 2.5)	Union (15 weeks) Healing extramedullary 12 weeks, intramedullary 21 weeks	3 avulsion proximal part, 2 superior migration; 1 displacement; 1 skin necrosis; 10 post-operation manipulation; 5 screws removal	Bicortical screws/Luque wires	IV
Chalidis [69]	2014	retrospective	53	39 months	Flexion-extension (7–85.6 to 1.87–106.75); ROM (78.8 to 104.88); KSS (40 to 80.4); KSF (35 to 65); WOMAC (43.54 to 17.52)	FTA (11 to 3.75); all but one united 16.7 weeks	Poor wound healing, subsequent breakdown, 1 non-union; 1 infection; 1 proximal tibia stress fracture	Wire fixation	IV
Choi [68]	2012	retorspective	36	57 months	ROM (40 to 92); KSS (47 to 82); KSF (9 to 72)	Union 11 weeks 1st stage, 21 weeks 2nd stage; Insall–Salvati (1.18 to 1.08)	1 non-union of avulsion fragment; 5 proximal migration; 2 avulsion fracture; 2 arthrofibrosis; 1 tibial shaft fracture;10 recurrent infections	3–5 wires (3 patients 2 wires + 2 cancellous screws)	IV
Chun [57]	2019	retrospective	31	5.2 years	KSS (85); HSS (83); ROM (101); flexion contracture (4)	Union 11.8 weeks; FTA (0.1); IS (0.8)	none	3.5 mm half-threated screws	III
Eid [76]	2016	prospective	20	2.5 years	HSS (46 to 85)	Union 21/23 in 4.5 months	1 deep venous thrombosis; 2 nonunion	3 bicortical screws	IV
Fletcher [60]	2015	case	1	3 months	ROM (0/5/105)	Union 3 months	none	screw	IV
Hay [49]	2010	prospective	32	2 years	VAS (9.6), WOMAC (47.2 to 3.9), KSS (~80); flexion (110.7 to 121.9)	X-ray	1 displacement od tibial tubercule,	2 × 3.5 mm cortical screws	II
Hirschmann [50]	2010	prospective	76	2 years	KSS (50 to 93), KSF (53 to 89); VAS (6.9 to 0.9); ROM (112 to 118)	Total knee arthroplasty roentgenographic evaluation and scoring system (TKA-RESS)	1 tibial plateau fracture, 2 secondarily displaced tibial tubercule	2 screws	III
Langen [51]	2015	retrospective	106	12 months	WOMAC (50.5 to 11.5), KSS (45.3 to 90), KSF (58.6 to77.2); ROM (111.9 to 115.9)	TFA (165.7 to 174.3)	3 additional transverse screw due to fracture of anterior tibial plateau, soft tissue revision	2 screws	III
Maruyama [59]	1996	case	3	2 years	ROM	Union (6 months)	none	Cortical/cancellous screws	IV
Mendes [67]	2004	retrospective	64	30 months	KSS (86)	Union (62/67)	2 nonunion; 2 extensor lag, 1 tibial fracture	Wires/wires + screws	IV
Le Moulec [75]	2014	retrospective	65	27.8 months	KSS (49.5 to 76.9); KSF (40.1 to 58.6); ROM (87.8 to 103.7)	Union 59/63 (16.9 months)	4 fragment migration	Cable wire	IV
Nakajima [61]	2010	case	1	1 year	-	FTA (179 to 171); Q angle (7.5 to 3.8);	None	2 cancellous srews	IV
Nikolopoulos [52]	2011	prospective	22	7 years	KSS (38.5 to 89.5), KSF (37.5 to 80); VAS (4 to 9), ROM (75 to 110)	Anatomical axis (23.5 to 5)	1 migration of tibial tubercule, 1 deep venus thrombosis	2 wires loop, in 2 patients 2 screws	I
Piedade [53]	2008	prospective	126	31.8 months	KSS (44 to 91); KSF (54 to 74), ROM (114 to 118)	Blackburne-Peel (0.8 to 0.7)	2 deep infection, 11 tibial plateau fractures,	2 screws	IV
Price [62]	2009	retrospective	5	29.7 months	KSS (70.5 to 85); ROM (93 to 101)	X-ray	cellulitis	4.5 lag screws	IV
Punwar [66]	2016	retrospective	38	2 years	ROM (85 to 95), OKS (16 to 29)	X-ray	2 proximal migration, 2 reinfection	Bicortical screw	IV
Ries [65]	1996	retrospective	29	18 months	-	X-ray	1 tibial tubercle fracture; 1 spiral tibia dipahysis	Titanium screws	IV
Schiapparelli [54]	2017	prospective	38	~3 years	none	FT axis (0.04)	Not reported	-	III
Segur [64]	2014	retrospective	26	3.4 years	KSS (59 to 78); KSF (51 to 70); WOMAC (55 to 88); ROM (90 to 95)	Union 22/26	2 non-union; 3 reinfections	Stainless steel wires/ethinbond suture	IV
Sun [55]	2014	prospective	27	50.9 months	KSS (93.4 to 126.2); HSS (42.2 to 71); WOMAC (56.3 to 38.4); flexion contracture (13.2 to 3.8); ROM (94.1)	FTA (0.7 to 0.1); IS (0.62 to 0.8)	2 partial patellar tendon avulsion; 1 periprosthetic deep infection	2 screws	III
Tabutin [63]	2011	retrospective	20	54 months	KSS (57.5 to 84); KSF (42.6 to 65); ROM (73 to 88)	Jacquot index (0.18 to 0.33)	1 nondisplaced tibia fracture; 2 stress fracture; 1 stiffness; 1 skin necrosis	2 screws	IV
Vandeputte [56]	2017	retrospective	13	2 years	KSS (73.73); KSF (53.46)	IS, BP ratio	none	Screws/wire	IV
Vives-Barquiel [74]	2015	retrospective	21	35 months	ROM (70 to 100); KSS (40 to 80); KSF (58 to 88); WOMAC (60 to 31)	BP (0.3 to 0.4); Portner angle (9 to 12)	3 lack of consolidation	Cerclage wire	IV
Young [73]	2008	retrospective	42	8 years	KSS (73 to 124) ROM (8–74 to 4–91)	Union 14 weeks	2 patella fracture	Luque wires	IV
Zonnenberg [72]	2014	retrospective	23	16.1 months	KSS (52.1); KSF (47.3); SF-36 (88)	Union	1 TTO fracture; 5 tibial plateau fractures	Absorbable sutures	IV

LOE—level of evidence; ROM—range of movement; VAS—visual analog scale, WOMAC—Western Ontario and McMaster Universities Osteoarthritis Index, KSS—Knee Society Score, KSF—Knee Society Score functional score, OKS—Oxford knee scale, HSS—Hospital for Special Surgery knee rating scale, FTA—femorotibial angle, HKA—hip–knee–ankle angle, Kujala—patellofemoral score, MAD—mechanical axis deviation, TT—tibial tubercle.

**Table 6:** Tibial shaft osteotomy around TKA.

Author	Year	Study Design	No. of Patients	Follow-Up	Clinical Outcomes	Radiological Outcomes	Complications	Fixation	LOE
Catonne [79]	2019	prospective	25	9 years	KSS (28.5 to 84); ROM (98 to 107)	HKA (180); TMA (74 to 89)	1 deep vein thrombosis; 4 secondary fracture line; 2 haematoma; 1 necrosis and infection; 1 extension lag	Screw/staple/plate	IV
Grzelecki [83]	2020	case	1	1 year	KSS (80); KSF (75); ROM (0–110)	union	none	-	IV
Hosokawa [84]	2017	case	1	2 years	ROM (0–125)	union	none	-	IV
Ishida [85]	2011	case	1	2 years	-	X-ray	Thermal necrosis	-	IV
Madelaine [81]	2014	retrospective	12	78 months	KSS (47.1 to 60.7); KSF (45.1 to 72.3)	mFTA (161.7 to 175.8)	4 tibial plateau fractures; 2 nonunion	Tibial base palte	IV
Radke [80]	2002	prospective	10	2.5 years	KSS (28 to 80.6); KSF (46.5 to 76)	IS (1.3)	none	screw	IV
Ramaswamy [82]	2009	retrospective	8	47.2 months	KSS (29.7 to 71.4); KSF (41.5 to 73.5); ROM (76.5 to 104.5)	Q angle (29.6 to 17.1)	1 flexion cantracture	Cancellous screws and washers	IV
Shibano [86]	2020	case	1	2 years	KSS (13 to 73); KSF (30 to 65); ROM (0–90)	FTA (135 to 178)	none	-	IV
Ucan [87]	2021	case	1	5 years	OKS (18 to 38); IKDC (19.2 to 52.9);	X-ray	none	tomofix	IV

LOE—level of evidence; ROM—range of movement, KSS—Knee Society Score, KSF—Knee Society Score functional score, OKS—oxford knee scale, FTA—femorotibial angle, HKA—hip–knee–ankle angle, TT—tibial tubercle.

## 4. Discussion

The purpose of our systematic review and meta-analysis was to evaluate the clinical, functional, and radiological results, as well as complication rates in TKA with the use of osteotomy simultaneously to optimize treatment effect. Osteotomies performed around the knee are technically challenging, but, in some cases, they are worthy procedures to consider. It may be the only option for treatment of extra-articular deformities and restoring proper alignment during TKA. The most important finding in this review is the utility of osteotomies in TKA. Every subgroup showed increases in functional and clinical outcomes. These outcomes were presented on various scales and scores (Table 7). Many authors used KSS/KSF and ROM. These scores are well-known, validated, and popular, especially for patients after TKA. ROM is a good reflection of post-surgery knee function. There is still a lack of RCTs in this topic, so the included studies are of low quality. Most of them have no control group, are retrospective, or are case series in nature.

**Table 7:** Quantity of scores in subgroups.

	KSS	KSF	ROM	Flexion Contracture	VAS	Kujala	IKDC	OKS	HSS	WOMAC
Distal femoral osteotomy	3	3	8	1		1		1	1	
Lateral condyle femoral osteotomy	5	3	3					2		1
Medial condyle femoral osteotomy	3	5	6	4				1	2	1
Medial tibial reduction osteotomy	2	2	1	1					1	
Tibial tubercle osteotomy	23	12	24	3	4			1	4	6
Tibial shaft osteotomy	6	5	5				1	1		
Total	42	30	47	9	4	1	1	6	8	8

ROM—range of movement, VAS—visual analog scale, WOMAC—Western Ontario and McMaster Universities Osteoarthritis Index, KSS—Knee Society Score, KSF—Knee Society Score functional score, AKSS—Knee Society Score activity score, OKS—oxford knee scale, HSS—Hospital for Special Surgery knee rating scale, Kujala—patellofemoral score.

All articles were finally split into six subgroups of osteotomy—femoral or tibial shaft, lateral or medial femoral condyle, medial tibial reduction, and tibial tubercule. The main criterion was the type of osteotomy. The extracted data allowed for the performance of a meta-analysis for only two—TTO and Medial femoral condyle osteotomy—among them. The TTO subgroup in the meta-analysis consisted of 590 patients, with a mean of 49 patients ranging from 13 to 126 patients. In every forest plot diagram, except one—VAS—the study with the biggest number of patients in the presented outcomes had the most important weighting. Langen et al. [51] had 106 patients included in the study in five main results (ROM, KSS, WOMAC, KSF, and FTA) with a mean weight of 50,34. Piedade et al. [53], who compared 126 patients in two outcomes (KSF and adverse events), had a mean weight of 46.35. Thus, in the future, we suggest performing more RCTs with a minimum of 100 patients in the control group in order to validate tibial tubercle osteotomy techniques in TKA. Additionally, those studies should be well designed and consistent, with a minimum of three scales or scores to effectively analyze the final results, according to the paper by Langen et al. [51], which was the most valuable due to multiple outcomes. However, such group sizes may not be possible in other subgroups. Medial and lateral side osteotomies include around 250 patients in every subgroup: a mean of 29 for lateral epicondyle femoral osteotomy, 44 for medial epicondyle femoral osteotomy, and 55 for medial tibial reduction osteotomy. We suggest a minimum sample size of 50 patients to validate those osteotomy techniques and with a heterogeneity of scales for presenting outcomes. Most cases consist of both tibial and femoral shaft osteotomies—five of eight in femoral osteotomy, and five of nine in tibial osteotomy. In those subgroups, even smaller sample sizes and well-designed RCTs could generate preliminary results. All studies were assessed by risk of bias. Further research with lower risk of bias is needed.

The most frequent type of osteotomy around TKA was TTO. Our review included 33 studies with 1382 patients. The results are promising for the pooled studies in the meta-analysis and the studies in the narrative review—increased from baseline clinical and functional scores when compared with control groups for both primary and revision TKA. Most of the studies described TTO as an approach for stiff knee or in revision TKA; only a few were treated for patella dislocation. Various outcome scores were used, including KSS, VAS, WOMAC, HSS, and OKS. None of the studies compared different types of fixations. The complication rate was significantly higher for the TTO group compared with the control group. In our opinion, more RCTs are needed. Future authors should consider a comparison of different types of fixations and aim to define the method with the lowest number of unwanted events—malunion, proximal distraction, reinfection, or fractures. More studies are also needed for the management of patella dislocation in TKA, both in primary and revision TKA.

Zonnenberg et al. performed a review of the literature in 2010 concerning TTO in primary and revision TKA evaluating clinical results and complications rates. Meta-analysis was not possible for them, because of different outcomes measures and inclusion criteria, as well as varied definitions of complication between authors. Their study included 823 knees [95]. After over 10 years of publishing, this number has increased—our study included 1382 knees, and the number of patients ranged from 1 to 159. They recommend standardized surgical techniques: the use of osteotome, except oscillation saw, cerclage wires rather than screws, and perform medial TTP with periosteal flap of 4–8 cm length [95]. In our opinion, there is still a strong need for RTC to clearly state recommendations in both primary and revision procedures, as well as septic and aseptic TKA.

In a systematic review performed by Divano et al., the authors tried to find the answer for two questions: How can TTO improve clinical outcomes? What is the safety and the rate of complications of TTO? First of all, TTO achieves satisfactory clinical and radiological results and improved exposure when it is not possible to retract the patella in 90 degrees. They suggest using TTO in primary TKA in two cases: rheumatoid arthritis and severe valgus deformity. The most common complications that should be considered are instability, loosening, malalignment, wound healing problem, postoperative motion deficit, patella stress fracture, and patella tracking problems. They conclude that most studies showed a reduction in the rate of these complications. They also noted that the use of TTO in revision TKA did not statistically influence the outcomes. Moreover, in two-stage revision, TKA provides superior clinical outcomes compared to other approaches. They also concluded, 8 years later than the Zonnenberg review, that there is still a need for longer studies with a larger group of patients [96]. Chalidis et al., in another review, considered TTO union as the primary outcome. They reported a 98.1% union rate—only 9 from 593 TTOs included in the analysis were not healed. Another complication was proximal migration—6.9%, 0.5% intraoperative tibial fracture, 1.7% proximal avulsion fracture, and 0.8% metaphyseal tibial fracture. Secondary outcomes were increased: ROM increased from 73.4 to 97, and knee flexion increased from 82.9 to 100.1. Only 27 patients required manipulation under anesthesia because of stiffness (4.6%). They recommend the usage of TTO, but also concluded that there is a strong need for RCT to standardize the protocol of performing TTO [97].

Our study confirmed all previous results, but there is still a lack of good-quality randomized prospective trials comparing TTO with different control groups and trying to find the proper answer about which technique is better. Screws, cerclage wires, or just sutures? We still require guidelines for rehabilitation protocols. There are also only a few studies concerning patella maltracking in primary and revision TKA. 

On the other hand, Baldini et al., in their study review, analyzed deformities around the knee in TKA: patients with many previous incisions, severe coronal deformity, genu recurvatum, stiff knee, extraarticular deformities, post-tibial or -femoral osteotomy, and neglected patellar dislocation. Some parts of the review also concerned osteotomies and made similar conclusions to our review. Deformities of distal femur or proximal tibia may require simultaneously corrective osteotomy with TKA to restore proper alignment. Before the procedure, the surgeon should consider intra-articular or extra-articular correction, the method of fixation (plate, intramedullary nail), and new techniques (computer assisted surgery) [98]. Femoral or tibial shaft osteotomies, as an addition for TKA procedures, are technically demanding but could be very useful, mostly for patients with extra-articular deformities, to achieve better outcomes. Our study showed, both for tibia and femur, that simultaneous correction of bone deformities leads to a significant increase in clinical and functional outcomes; however, there is a lack of studies concerning this problem and RCT, probably due to a low number of patients with severe extra-articular deformities. Besides this, proper guidelines after well-designed studies will be useful in managing those patients in the future.

Varus and valgus knees are very common, and surgeons should consider whether a standard TKA procedure will be sufficient to restore alignment. Good preoperative planning, including radiological and clinical tests, is necessary to assess collateral ligament efficiency. Correcting valgus knee deformity >1st grade (>10 degrees) is a technically challenging procedure because of stretch medial collateral structures and contracted lateral complex (iliotibial band, posterolateral capsule, lateral collateral ligament, and popliteus tendon). To achieve good clinical outcomes, well-balanced knee replacement is necessary. Soft tissue balancing may be insufficient in severe deformity. If deformity cannot be corrected, other procedures should be considered, such as correction osteotomies. Our review showed that lateral femoral condyle osteotomy is a good option with improved outcomes in the last follow-up. Moreover, medial epicondylar osteotomy is a good alternative method for pathologic contracture of medial soft tissue for both varus and valgus deformities. This procedure is technically simple and does not cause massive damage. However, differences for studies included in meta-analysis were not significantly in favor of the experimental group, but overall, prospective and retrospective studies about this type of osteotomy presented improved clinical and functional outcomes compared to preoperative. In addition, medial tibial reduction could be applied for the correction of varus knee in TKA. We conclude that there is also a strong need for more well-designed studies.

Tibial or femoral shaft osteotomies are quite rare. They are performed only in severe extra-articular deformities for proper correction and to restore alignment. Posttraumatic malformation or complications after surgery, such as nonunion or overcorrection, are the main causes of additional shaft osteotomy in TKA. As mentioned before, half of the presented studies were cases, but still both subgroups presented improved outcomes. RCTs with large groups of patients may be impossible to arrange. However, even retrospective or prospective studies with a small group of patients could confirm the adequacy of tibial/femoral shaft osteotomy with TKA.

Some studies also presented outcomes in X-rays, measuring specific angles after restoring alignment and patella ratios. For future studies, we strongly recommend measuring patients pre- and postoperatively, according to Paley et al. An analysis of those surveys and comparison with control groups could provide additional information concerning what advantages come from every kind of osteotomy and how even small corrections could improve outcomes.

### 4.1. Strengths

Our major strength is the inclusion of all kinds of osteotomies performed around the knee, in addition to TKA, as well as no timeframes for searching in databases. We also summarized the main information from studies in tables in order to make it easy to find any needed materials. Our study included more studies than any previous review. The results are quite promising and give direction for future studies.

### 4.2. Limitations

The main limitation of this review is the insufficiency in the number of RCTs. Only two subgroups could be included in the meta-analysis. Most of them also presented outcomes on different scales and scores, and the studies included have low quality. There are also many case series, and many trials had no control group, in addition to being retrospective. Twelve studies with a few domains at a high risk of bias could have influenced the final results. We also included only English language articles; however, only 12 articles were excluded due to this criterion.

## 5. Conclusions

Osteotomies performed around the knee simultaneously with TKA are useful and, in some cases, indispensable to restore proper lower extremity alignment. They should be well planned before surgery, according to the principles of deformity correction. Clinical, functional, and radiological outcomes improve when compared to the baseline and control groups; however, multiple RCTs should be performed before we are able to state a clear indication for osteotomy concomitant with TKA. This is a procedure that would create a significant difference in outcomes in favor of the osteotomy groups.
